# Vitrectomy with and without encircling band for pseudophakic retinal detachment with inferior breaks: VIPER Study Report No. 3

**DOI:** 10.1007/s00417-018-4106-6

**Published:** 2018-08-23

**Authors:** Sabine Baumgarten, Petra Schiller, Martin Hellmich, Peter Walter, Hansjürgen Agostini, Bernd Junker, Horst Helbig, Albrecht Lommatzsch, Babac Mazinani, on behalf of the VIPER Study Group, P. Walter, B. Mazinani, S. Baumgarten, P. Schiller, M. Hellmich, G. Rössler, H. Agostini, B. Junker, H. Helbig, A. Lommatzsch, F. Holz, B. Kirchhof, E. Limburg, K.-U. Bartz-Schmidt, A. Joussen, S. Aisenbrey, M. Partsch, T. Neß, A. Pielen, C. Dahlke, S. Fauser, A. Lappa, N. Eter, C. Uhlig, U. Ritzau-Tondrow, N. Feltgen, M. A. Gamulescu, M. Rudolf, M. Lüke, M. Gök, P. Wiedemann, C. Jochmann, P. Meier, A. Nestler, W. Rasche, C. Clemens, J. Hillenkamp, H. Roider

**Affiliations:** 1 Department of Ophthalmology, RWTH Aachen University, Pauwelsstr. 30, 52074 Aachen, Germany; 2 Institute of Medical Statistics and Computational Biology, University of Cologne, Cologne, Germany; 3 Eye Center, Albert-Ludwigs-University of Freiburg, Freiburg im Breisgau, Germany; 4 University Eye Hospital, Hannover Medical School, Hannover, Germany; 5 Department of Ophthalmology, University of Regensburg, Regensburg, Germany; 6 Department of Ophthalmology, Franziskus Hospital, Münster, Germany

**Keywords:** Inferior breaks, Pseudophakic, Randomized clinical trial, Retinal detachment, Scleral buckling, Vitrectomy

## Abstract

**Purpose:**

To test if an encircling band improves outcomes in vitrectomy for pseudophakic retinal detachment (PRD) with inferior or with multiple (4 or more) breaks.

**Methods:**

Subgroup analysis of a prospective randomized controlled multicenter trial in patients with uncomplicated PRD assigned either to 20 G vitrectomy plus encircling band (group E1), or 20 G vitrectomy without any buckle (group C), or 23/25 G vitrectomy without any buckle (group E2). The primary endpoint was defined as no indication for any retina reattaching procedure during the review period of 6 months. One hundred out of 257 patients were identified with inferior breaks and 63 patients had 4 or more breaks.

**Results:**

In patients with retinal breaks between 5:00 and 7:00, treatment was successful in 77.4% (24/31, treatment arm E1) versus 57.1% (16/28, treatment arm C) (*p* = 0.301, odds ratio (OR) 1.83, 95% confidence interval (CI) 0.48 to 7.17). In patients with multiple breaks, success rates were 68.2% (15/22, E1) versus. 72.4% (21/29, C, *p* = 0.46, OR 0.52, CI 0.08–3.65).

**Conclusion:**

Combining an encircling band with vitrectomy in patients with pseudophakic retinal detachment and inferior or multiple breaks does not significantly improve primary anatomical success in comparison to treatment with 20 G or 23/25 G vitrectomy alone.

## Introduction

Retinal detachment with inferior breaks or multiple breaks may be associated with unfavorable success when treated with vitrectomy and gas alone due to reduced support of the inferior breaks by the endotamponade. Accordingly, increased anatomical and functional failure has been reported in such cases [1–3]. The prospective randomized controlled VIPER study showed that an additional encircling band (EB) does not significantly reduce the risk of any second procedure necessary to reattach the retina in patients with primary pseudophakic retinal detachment [4, 5]. However, an additional buckle may still be helpful in patients with inferior or multiple breaks. The aim of this post hoc analysis was to test if an additional buckle increases anatomical success compared to 20 G vitrectomy without additional buckle in the subgroup of patients with inferior breaks as well as in the subgroup of patients with multiple breaks (4 or more breaks).

## Methods

The VIPER Study (*Vi*trectomy with and without encircling band in the treatment of *p*s*e*udophakic *r*etinal detachment) was a prospective, multicenter, randomized clinical trial registered in the German Register for Clinical Trials (DRKS 00003158, www.germanctr.de). A total of 34 vitreoretinal surgeons in 14 clinical sites across Germany participated. The study protocol was approved by each local ethics committee. Details of the study methods and the primary analysis have been described in a design publication [5]. Briefly, 257 patients with uncomplicated pseudophakic retinal detachment were randomized to the following groups:

- Control group (C): In this group, patients received a 20 G vitrectomy without encircling band. Surgery started with opening the conjunctiva at the limbus to uncover the sclera. With a distance of 3–4 mm to the limbus, three 20 G sclerotomies were made, and subsequently, a full vitrectomy was performed. A complete vitreous detachment could be realized if the vitreous was not fully detached. Subretinal fluid was drained through heavy liquids. Retinal breaks and high-risk degenerations were treated with endolaser or transconjunctival/transscleral cryopexy once the retina was fully reattached under air or heavy liquids. After full fluid-air exchange, a gas fill using non-expandable air/gas mixtures such as SF6, C2F6, or C3F8 completed surgery. The sclerotomies and the conjunctiva were sutured and ocular pressure was monitored within 8 h after surgery and the day after surgery.
- Experimental group 1 (E1): Patients received a 20 G vitrectomy with an encircling band. The conjunctiva was opened circumferentially at the limbus and a 2–4-mm encircling band was positioned underneath the recti muscles and subsequently fixated in all four quadrants onto the equator of the globe. Adequate buckling with no choroidal folds should be achieved at the end of surgery. Following, the 20 G vitrectomy was executed as described above for the control group (C).
- Experimental group 2 (E2): In this group, patients received a 23 G or 25 G vitrectomy without encircling band. The conjunctiva was shifted and valved or unvalved transconjunctival trocar systems were inserted tangentially. A full vitrectomy was performed and a full vitreous detachment achieved. Subretinal fluid was drained through the use of heavy liquids and/or air. Retinal breaks and high-risk degenerations were treated with endolaser or transconjunctival/transscleral cryopexy. After full fluid-air exchange, a non-expandable air/gas mixture was inserted. At the end of surgery, trocars were removed and sclerotomies were sutured when leaking.
- All groups (C, E1, E2): Surgery was executed under an operating microscope and a wide field viewing system (contact/non-contact). The use of triamcinolone, silicon oil, or internal limiting membrane peeling was not allowed as well as a prophylactic laser treatment.

Dependent on surgical skills of the individual surgeon, patients were randomized either with ratio 1:1 between E1 and C, or with ratios 1:1:1 between E1, C, and E2.

The primary endpoint was defined as no indication for any procedure to reattach the retina during the follow-up of 26 weeks. A missing primary endpoint was counted as failure.

For the post hoc analysis, the information on preoperative breaks recorded by the participating surgeons was verified and updated by an evaluation committee (SB, BM). Five different subgroups were compared: Patients with breaks (1) at the 6:00 position, (2) between 5:00 and 7:00, (3) between 4:00 and 8:00 and (4) in the lower hemisphere that is between 3:00 and 9:00, and (5) patients with 4 or more breaks regardless of the localization.

The analysis of the treatment success in the subgroups was based on all subjects, who were randomized and who received surgery. Patients were analyzed for the treatment group to which they were assigned (intention-to-treat principle).

The comparison of E1 versus C regarding the primary endpoint, absence of indication for reattaching procedure, was evaluated using the Cochran-Mantel-Haenszel test stratified by surgeon and the corresponding common odds ratio (OR) [6]. Analyses are essentially descriptive; thus, no correction for multiple testing was applied. Statistical analyses were done with SPSS Statistics software (version 13, IBM Corp., Armonk, NY, USA).

## Results

Thirty-eight out of 257 patients (14.8%; C, 13; E1, 16; E2, 9) had breaks at the 6:00 position and were included in group 1. Seventy-two patients (28%; treatment arm C, 31; E1, 28; E2, 13) were included in group 2 with breaks between 5:00 and 7:00, 94 patients (36.6%; C, 37; E1, 39; E2, 18) were included in group 3 and had breaks between 4:00 and 8:00, and 100 patients (38.9%; C,41; E1, 41; E2, 18) were included in group 4 with breaks in the lower hemisphere.

Sixty-three patients (24.5%; C, 22; E1, 29; E2, 12) had 4 or more breaks and were included in group 5. Table 1 shows as example the baseline characteristics of patients with inferior breaks between 5:00 and 7:00 in the treatment arm C compared to E1. The preoperative characteristics were fairly balanced among the treatment arms.

**Table 1:** Preoperative characteristics of patients in subgroup with breaks between 5:00 and 7:00

Characteristic	Surgery	
	(E1) 20 G vitrectomy with EB (*n* = 31)	(C) 20 G vitrectomy without EB (*n* = 28)
	Summary statistics	Summary statistics
Sex, male	23 (74.2%)	19 (67.9%)
Age (years)	65 ± 9	66 ± 10
Study eye, right	19 (61.3%)	11 (39.3%)
Sphere (diopter)^‡^	0.25 (0.00 to 1.00)	0.00 (− 1.25 to 0.00)
Cylinder (diopter)^‡^	− 0.50 (− 1.25 to 0.00)	− 1.00 (− 1.00 to 0.00)
Axis (degree)^‡^	40 (0 to 140)	57 (0 to 110)
Intraocular pressure (mmHg)^‡^	14 (12 to 17)	15 (12 to 18)
Visual acuity, logMAR^‡^	1.6 (0.8 to 1.7)	0.7 (0.2 to 1.7)
Vitreous situation at start of surgery		
Fully attached	2 (6.5%)	2 (7.1%)
Partly attached	9 (29.0%)	7 (25.0%)
Fully detached	19 (61.3%)	17 (60.7%)
Hemorrhage	0 (0.0%)	1 (3.6%)
Other	1 (3.2%)	1 (3.6%)

^‡^Percentage of missing data ≤ 3.6%; otherwise complete data

**Fig. 1:**
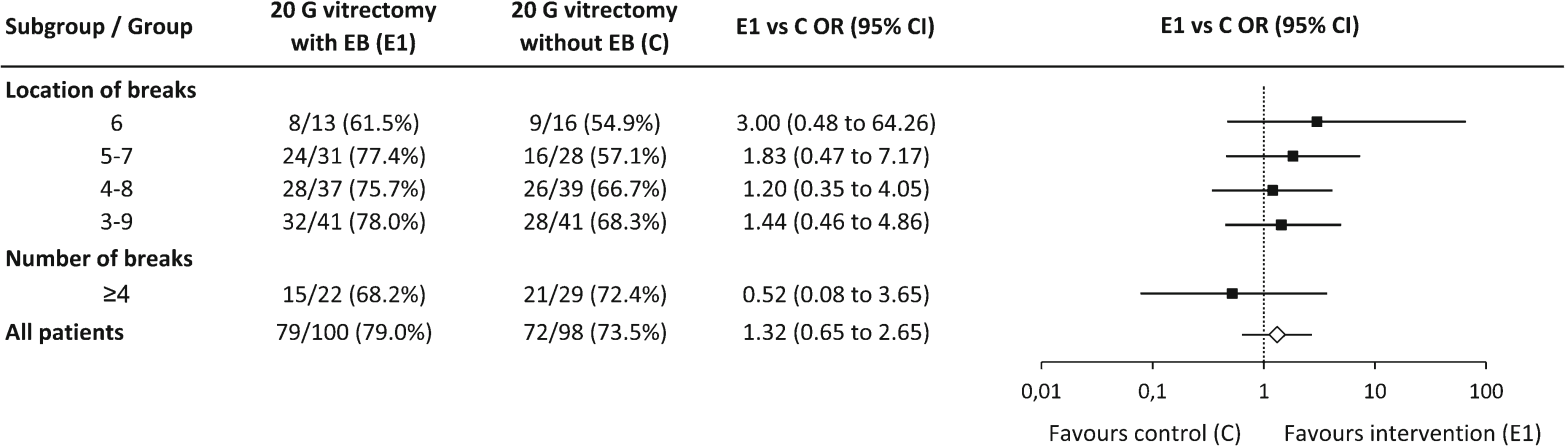
Primary outcome analysis with respect to the subgroups. (Primary outcome is defined as absence of indication for reattaching procedure; thus, the odds ratio (OR) states the chance of achieving a successful outcome after vitrectomy with EB (E1) compared to vitrectomy without EB (C), CI confidence interval.)

Considering all patients regardless of the location of breaks, the primary anatomical success rate was 79% in the arm treated with 20 G vitrectomy plus an encircling band and 73.5% in the control arm without encircling band (OR 1.32, CI 0.65 to 2.65; Fig. 1) [7]. If only patients with inferior breaks are considered, the percentage with an anatomical success was higher in the treatment arm with encircling band compared to control (for location of breaks 3-9: 78% with EB vs. 68.3% without EB; location of breaks 4-8: 75.7% vs. 66.7%; Fig. 1). This difference was most distinct in patients with breaks between 5:00 and 7:00 with 77.4% (24/31, treatment arm E1) versus 57.1% (16/28, treatment arm C). The odds ratio (E1 with regard to C) was 1.83 (95% confidence interval (CI) 0.48 to 7.17, *p* = 0.301). However, in none of the comparisons, the odds ratio differed significantly from 1.

In patients with 4 or more breaks, the respective success rates were 68.2% (15/22, treatment arm E1) versus 72.4% (21/29, treatment arm C, odds ratio 0.52, 95% confidence interval 0.08 to 3.65, *p* = 0.46; Fig. 1).

## Discussion

Whereas some authors have reported favorable results in treating retinal detachments with inferior breaks by vitrectomy alone [8, 9], others found superior primary attachment rates in their patients treated with vitrectomy plus buckling compared to published results without additional buckle [10]. To our knowledge, the only study comparing the two treatments in retinal detachment with inferior breaks using a control group is a retrospective study by Wickham et al. [11]. The authors reported on 86 patients with retinal detachment and inferior breaks with a primary anatomical success rate of 89% if treated with vitrectomy and gas and 73% if treated with vitrectomy and additional buckle without significant difference. Because of the retrospective design of the study, bias in the assignment of the treatment may be assumed. Accordingly, the authors report a higher rate of proliferative vitreoretinopathy in the combined treatment group (20% vs. 5%).

In our investigation, we found that the anatomical success was higher in patients with inferior breaks when treated with an additional encircling band compared to the control group without EB. This trend was most pronounced in group 2 with breaks between 5:00 and 7:00 (77.4% vs. 57.1%). The odds ratio of 1.83 indicates an increasing chance of success if an encircling band is added as compared to control, though the results were not significant. By including breaks at more superior locations in the inferior hemisphere, the need for additional buckling may be reduced by a better effect of the endotamponade. When focusing on breaks at the 6:00 position (group 1), where the endotamponade can be assumed least effective and therefore an additional buckle most helpful, only 38 patients could be included in the present analysis which may account for the less distinct results compared to group 2. Our results show a trend that the combined procedure with vitrectomy and encircling band in pseudophakic retinal detachments with inferior breaks might be beneficial. However, improvement of the outcome was not statistically significant. In patients with multiple (4 or more) breaks regardless of the position, the success is similar in both treatment groups.

Our study is limited by the relatively small number of included subjects due to focusing on cases with inferior or multiple breaks, which may account for the failure to show a significant effect of additional buckling. The low success rates in any groups are because of the complicate cases of retinal detachments. Having inferior or multiple breaks provides a difficult starting position. In Viper Report No. 2, we reported on our results regardless of the break position. Success rates here were higher (E1, 79% vs. C, 73.5% and E2, 87.7% vs. C, 78.7% [7]). To the best of our knowledge, this is the only study that excluded bias in treatment assignment due to the randomized controlled study design.
